# Correction to “Serum‐Free Culture System for Spontaneous Human Mesenchymal Stem Cell Spheroid Formation”

**DOI:** 10.1155/sci/9895609

**Published:** 2026-02-23

G. Dong, S. Wang, Y. Ge, et al., “Serum‐Free Culture System for Spontaneous Human Mesenchymal Stem Cell Spheroid Formation,” *Stem Cells International* 2019, no. 1 (2019): 6041816, https://doi.org/10.1155/2019/6041816.

In the article, the two upper Day 3 panels of Figure 1C,D share unexpectedly repeated elements to the p5‐Day 3 panel of Figure 2b, initially identified on PubPeer [1]. The authors have clarified that this was due to an error introduced during the figure preparation. The corrected Figure 1 is as follows:

Figure 1MSCs at P3 can spontaneously form spheroids in medium containing KSR. (A) Schematic diagram shows the experimental procedure; (B) MSCs cultured in MiPS and MiPS without KSR/GLn/NEAA/βME/bFGF; (C, D) MSCs spheroids was generated and stained with Calcein‐AM /PI in MiPS/KSR at Days 1, 3 and 6 on tissue culture dishes. Statistical analysis of mean diameter and cell viabilities of MSC spheroids. Scale bars: 100 µm.

**Figure:**
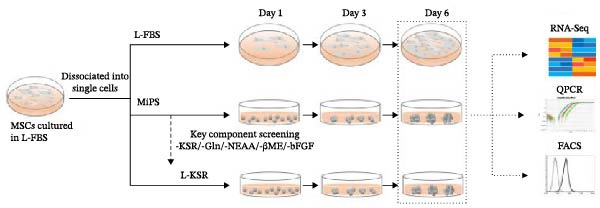
(A)

**Figure:**
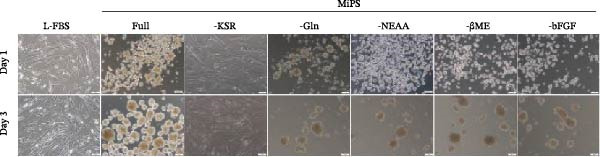
(B)

**Figure:**
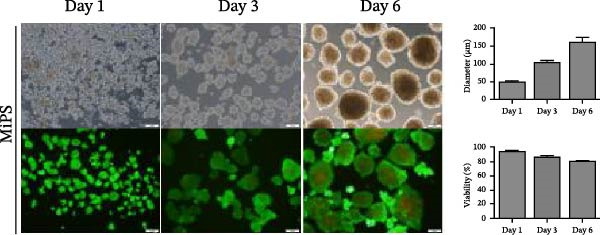
(C)

**Figure:**
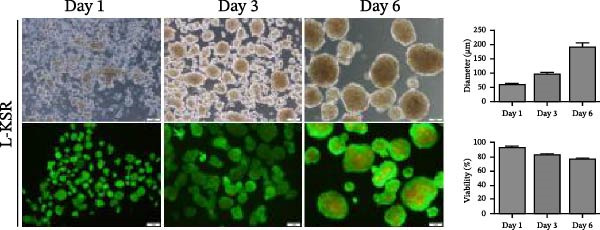
(D)

Additionally, the conflicts of interest statement should be updated to the below:

“Several authors are affiliated to BGI Shenzhen (BGI Research‐Shenzhen), a non‐profit research institution focused on scientific exploration and discovery, without taking industrialization or commercialization as its goals. Neither the technical methods described in this article nor developments from this work are associated with any related patents. Therefore, no conflict of interest exists.”

We apologize for these errors.
