# Supplementary material for: Serum-Free Culture System for Spontaneous Human Mesenchymal Stem Cell Spheroid Formation
Source: Stem Cells Int. 2019 Oct 15;2019:6041816. doi: 10.1155/2019/6041816 (PMC6815607; doi:10.1155/2019/6041816)
Supplement: Supplementary 1 — Figure S1: MSCs can form spheroids in KSR-contained medium. (A) hMSCs cultured in L-FBS at passage 3; (B) hMSC spheroids at day 6 generated from hMSCs at passage 3 in L-DMEM at the indicated concentration KSR. Statistical analysis of the hMSC spheroid mean diameter cultured in different concentrations of KSR in L-DMEM medium; (C) hMSC spheroids at day 6 generated from hMSCs at passage 3 in various basal media, including RPMI1640, DMEM/F12, H-DMEM, and MEM with 20% KSR. Statistical analysis of the hMSC spheroid mean diameter cultured in various basal media with 20% KSR. Scale bars: 100 μm. [file 6041816.f1.pdf]

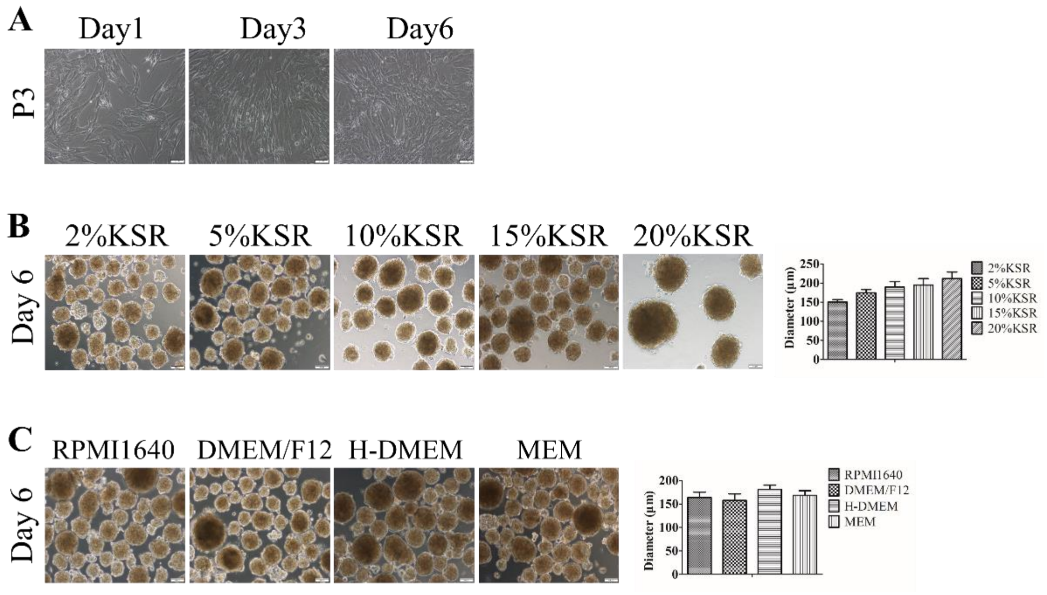

Figure S1 MSC cells form spheroids in KSR containing medium.

(A) Morphology of hMSCs cultured in L-FBS at passage 3 at day 1, 3 and 6; **(B)** hMSC spheroids at day 6 generated from hMSCs at passage 3 in L-DMEM at the indicated concentrations of KSR. Statistical analysis of spheroids diameter of hMSCs cultured in different concentrations of KSR in L-DMEM medium; **(C)** hMSC spheroids at day 6 generated from hMSCs at passage 3 in different basal medium, including RPMI1640, DMEM/F12, H-DMEM, MEM, with 20% KSR. Statistical analysis of spheroids diameter cultured of hMSCs cultured in different medium system. Spheroid diameters were measured from captured images (n = 12-20), and values show mean ± SD (n = 3). Scale bars:100 μm
