# Supplementary material for: Serum-Free Culture System for Spontaneous Human Mesenchymal Stem Cell Spheroid Formation
Source: Stem Cells Int. 2019 Oct 15;2019:6041816. doi: 10.1155/2019/6041816 (PMC6815607; doi:10.1155/2019/6041816)
Supplement: Supplementary 2 — Figure S2: MSCs can form spheroids at different cell concentrations in 20% KSR-containing medium. (A) hMSC spheroids generated from MSCs at passage 3 in L-DMEM with 20% KSR at different cell concentrations; (B) statistical analysis of the hMSC spheroid mean diameter cultured in different concentrations of KSR in L-DMEM medium. Scale bars: 100 μm. [file 6041816.f2.pdf]

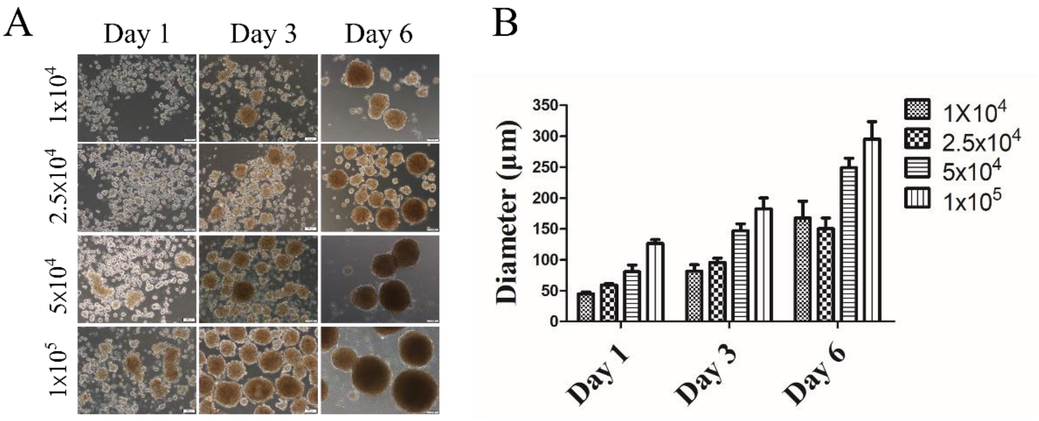

Figure S2 MSCs form spheroids at different cell concentrations in 20% KSR containing medium.

(A) hMSC spheroids generated from MSCs at passage 3 in L-DMEM with 20% KSR at different cell concentrations; **(B)** Statistic analysis of hMSC spheroids diameter cultured in different concentrations of KSR in L-DMEM medium. Spheroid diameters were measured from captured images (n = 12-20), and values show mean ± SD (n = 3). Scale bars: 100 μm
